# Pleiotropic effect of a novel mutation in *GCNT2* causing congenital cataract and a rare adult i blood group phenotype

**DOI:** 10.1038/hgv.2017.4

**Published:** 2017-02-16

**Authors:** Sek-Shir Cheong, Sarah Hull, Benjamin Jones, Ravinder Chana, Nicole Thornton, Vincent Plagnol, Anthony T Moore, Alison J Hardcastle

**Affiliations:** 1 UCL Institute of Ophthalmology, London, UK; 2 Moorfields Eye Hospital, London, UK; 3 IBGRL Red Cell Reference Laboratory, NHS Blood and Transplant, Bristol, UK; 4 UCL Genetics Institute, London, UK; 5 Ophthalmology Department, UCSF School of Medicine, San Francisco, CA, USA

## Abstract

Mutations in *GCNT2* have been associated with the rare adult i blood group phenotype with or without congenital cataract. We report a novel homozygous frameshift mutation c.1163_1166delATCA, p.(Asn388Argfs*20) as the cause of congenital cataract in two affected siblings. Blood group typing confirmed that both affected males have the rare adult i phenotype, supporting the hypothesis that the partial association of I/i phenotype and congenital cataract is due to the differential expression of *GCNT2* isoforms.

Congenital cataracts (CCs) account for 3–5% of visual impairment in children in the United Kingdom,^1^ with a prevalence of 1–6 per 10,000 births, at least 50% of which are inherited.^2,3^

CC can be inherited as an isolated phenotype, in combination with other ocular features including microphthalmia/anophthalmia and aniridia,^4,5^ or as a syndromic condition associated with a broad range of extra-ocular phenotypes, such as developmental delay, skeletal defects and dental anomalies.^6,7^ Identification of the genetic cause of CCs is challenging due to genetic heterogeneity,^2^ and in some cases establishing genotype–phenotype correlation is hindered by intrafamilial phenotypic variability and variable disease progression.^7,8^

The human blood group I and i antigens are carbohydrate structures on glycoproteins and glycolipids on the cell surface, which were first discovered on human red blood cells.^9^ These antigens were subsequently identified in other tissues including reticulocytes and lens epithelium.^10^ The phenotype of I/i blood group is determined by the presence of I or i antigens and the expression of these antigens is developmentally regulated; i antigens are predominant on fetal red blood cells, whereas adult human red blood cells fully express I antigens with a very low level of i antigens. The conversion of i to I occurs during the first 18 months after birth as a result of the expression of the I-branching enzyme, β-1,6-*N*-acetylglucosaminyltransferase 2 (encoded by *GCNT2*), which adds a GlcNAc-β-1–6 branch onto the poly-GlcNAc chains.^11,12^ Therefore, absence of this enzyme gives rise to the adult i phenotype, a rare autosomal recessive condition.^13,14^

The association of recessive mutations in the *GCNT2* gene with CC and the rare adult i phenotype has been reported in 11 families, of differing ethnic origin (Table 1).^10,15–19^ In this study, we identified a novel homozygous *GCNT2* frameshift mutation in a reportedly non-consanguineous Caucasian family with CC by whole-exome sequencing (WES), and subsequent I and i blood typing confirmed an adult i phenotype.

A reportedly non-consanguineous three-generation Caucasian family comprising two affected brothers, II:2 and II:3, was recruited to the study (Figure 1a). Patient (II:2), now age 40 years, was noted in early infancy to have reduced vision. Examination under anesthesia was performed at the age of 4 months, which identified pendular nystagmus and bilateral lamellar cataracts. Initial management was with pupil dilatation using guttae atropine 0.5% in each eye. At 10 months of age, cataract surgery was performed with lens aspiration, which left him aphakic. Contact lens refractive correction was subsequently used. Further treatment included two left lens surgical capsulotomies at the age of 2 and 17 years, right occlusion therapy for left amblyopia and squint surgery for left esotropia at the age of 2 years. At the age of 3 years, the first recorded uniocular visual acuity was 6/24 Snellen (right eye, RE), and 4/60 (left eye, LE). At last review, at 40 years old, visual acuity was 6/60 (RE), and 6/24 (LE) with refractive correction of +13.25/−2.25×105 (RE), +14.75/−1.25×170 (LE).

Patient (II:3), now age 39 years, was noted at 2 months of age by his mother to have nystagmus and a white reflex. Central lens opacities were found with abnormal posterior curvature of the lens, and normal fundi. Lens aspiration was performed in the left eye at 7 months of age, and in the right eye at 10 months of age with soft contact lens refractive correction afterwards. Further procedures included left needle capsulotomy at 8 months of age, left laser capsulotomy at the age of 15 years, secondary sulcus intraocular lens in the right eye at the age of 19 years and in the left eye at 29 years of age. Axial lengths on B scan ultrasound prior to lens insertion demonstrated long axial lengths of 27.88 mm (RE), 29.31 mm (LE) and refractive errors of +10.50/−1.00×10 (RE), and +10.00/−2.00×15 (LE). At the age of 29 years, Snellen visual acuity was 6/36 in both eyes. Fundus examinations and electroretinogram were normal in both siblings and there was no evidence of anterior segment dysgeneses or glaucoma.

Both parents were examined. Their mother (I:2) had subtle lamellar lens opacities at the age of 61 years. However, given her age and prevalence of cataracts in the general population at that age, these findings could be age-related. Their deceased father (I:1) was unilaterally aphakic (trauma-related), the other lens was clear. Both affected siblings had uncomplicated births and were well with normal development. All investigations were conducted in accordance with the principles of the Declaration of Helsinki. The study was approved by the local ethics committees at Moorfields Eye Hospital, UK. After written informed consent was obtained from all subjects, blood samples were donated and genomic DNA was extracted from peripheral blood lymphocytes using conventional methodologies. Patients were clinically assessed by experienced ophthalmologists. Inheritance of CC was consistent with recessive disease (Figure 1a).

WES was performed for individual II:3 using Nimblegen sequence capture (v2) and a HiSeq2000 sequencer (Illumina, San Diego, CA, USA). Reads were aligned to the hg19 human reference sequence using Novoalign (Novocraft, www.novocraft.com) version 2.05. The ANNOVAR tool (OpenBioinformatics, www.openbioinformatics.org/annovar/) was used to annotate sequence variants. Filtering was performed to identify variants with a minor allele frequency ⩽0.005 in 1000 Genomes Project (www.1000genomes.org/), the National Heart, Lung, and Blood Institute Exome Sequencing Project Exome Variant Server (http://evs.gs.washington.edu/EVS/), Exome Aggregation Consortium database (http://exac.broadinstitute.org/) and our internal University College London exomes consortium database comprising of 1,980 exomes. Variants were then cross-referenced with CatMap (http://cat-map.wustl.edu/) for variants in known cataract genes. WES data were also analyzed by ExomeDepth^20^ to identify any potential causative exonic copy number variations. The *GCNT2* variant in exon 3 was tested for segregation in the affected males (II:2 and II:3), their mother (I:2) and the children of individual II:2 (III:1 and III:2) by direct sequencing. Primer sequences are available on request. *GCNT2* cDNA is numbered in accordance with Ensembl transcript ID ENST00000316170, with +1 corresponding to the A of the ATG translation initiation codon.

WES analysis of individual II:3 identified a unique homozygous 4-bp deletion in *GCNT2* (Figure 1b), predicted to cause a frameshift mutation, c.1163_1166delATCA, p.(Asn388Argfs*20). Copy number variation analysis of this WES data did not identify any potential exonic copy number variations in any genes associated with CC, and excluded copy number variations at the *GCNT2* locus. Direct sequencing of *GCNT2* exon 3 confirmed that both affected males are homozygous for the frameshift mutation, whereas their mother (I:2) and two children (III:1 and III:2) of affected male II:2 are carriers (Figure 1c).

*GCNT2* has three isoforms, *GCNT2-A*, *GCNT2-B* and *GCNT2-C*, which are alternatively spliced with a different exon 1 (refs 10,16) (Figure 2). These isoforms are differentially expressed, with only transcript *GCNT2-B* expressed in lens epithelial cells and *GCNT2-C* in reticulocytes. Differential expression of *GCNT2* isoforms has been proposed as a potential mechanism for the absence of CC in some patients with an adult i blood group.^10^

The mutation identified in this family is located in exon 3, and is therefore present in all three *GCNT2* isoforms (Figure 2), suggesting these individuals may also have an adult i blood group. Blood samples were collected in EDTA tubes for I/i blood group typing for individuals II:2 (age 40 years) and II:3 (age 39 years). Monoclonal anti-I (HIRO-245) from the Japanese Red Cross, and polyclonal anti-i (P.E.) from the in-house reference collection were tested by standard direct agglutination tube technique and scored according to Marsh.^21^ Expression of i antigen was assessed by titration, using a base dilution of 1:40 and then doubling dilutions. An example of adult cells with normal I expression (L2325-8) and adult i cells (074-214RF) from the in-house reference collection were included as controls.

Our finding confirmed that both affected individuals have the adult i phenotype, thereby establishing the association of the homozygous *GCNT2* frameshift mutation p.(Asn388Argfs*20) with CC and the adult i phenotype in this family.

Thus, in this study, we describe the first report of the association of *GCNT2* mutation with CC and rare adult i phenotype in the Caucasian population, and our data support the hypothesis that differentially expressed *GCNT2* isoforms account for the partial association of the adult i phenotype with CC, irrespective of ethnicity.

## Figures and Tables

**Figure 1:**
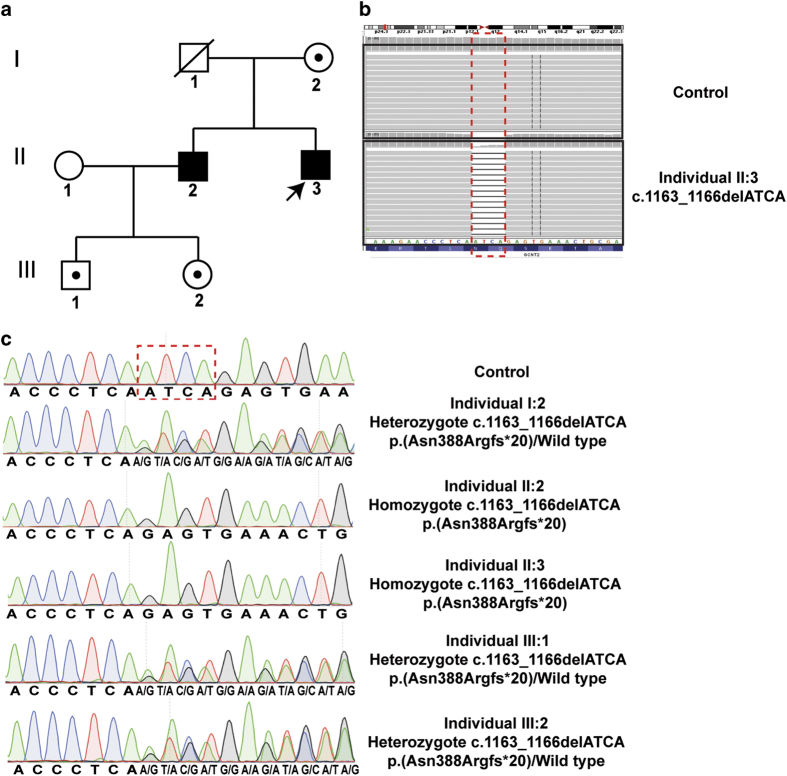
Novel homozygous *GCNT2* frameshift mutation in a CC family. (**a**) Pedigree of the study family with two affected siblings. Shaded squares denote affected males; dotted circles, carrier females; dotted square, carrier male. Arrowhead indicates proband in the family. (**b**) Exome sequence alignments of control (top panel) and individual II:3 (bottom panel) viewed using Integrative Genomics Viewer (https://www.broadinstitute.org/igv/) shows a 4-bp deletion in exon 3 of the *GCNT2* gene in the proband (indicated by dashed box). Nucleotide sequences and corresponding amino acid residues are shown below the exome data tracks. (**c**) Sequence electropherograms demonstrate segregation of the *GCNT2* mutation. The proband (II:3) and his affected brother (II:2) are homozygous for the 4-bp deletion. Their mother (I:2) and the children of II:2 (III:1 and III:2) are carriers for the mutation. Control sequence electropherogram is shown above I:2 sequence. The exon 3 mutation is predicted to cause a frameshift [c.1163_1166delATCA, p.(Asn388Argfs*20)]. *GCNT2* cDNA is numbered in accordance with Ensembl transcript ID ENST00000316170, with +1 corresponding to the A of the ATG translation initiation codon.

**Figure 2:**
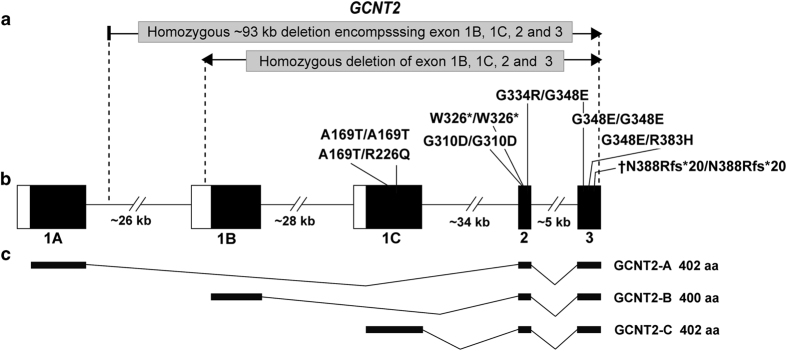
*GCNT2* gene structure and isoforms annotated with reported mutations. (**a**) All previously reported *GCNT2* mutations, including homozygous and compound heterozygous missense mutations, a homozygous nonsense mutation and segmental deletions.^10,15–19^ Mutations in *GCNT2* exon 2 or exon 3,^15–18^ or segmental deletions encompassing exons 1B, 1C, 2 and 3,^15,19^ were reported in patients with CC and an adult i blood group phenotype (Table 1), whereas the mutations in exon 1C (homozygous A169T/A169T and compound heterozygous A169T/R226Q) were found in patients with an adult i blood group phenotype without cataract^10^ (Table 1). The novel homozygous *GCNT2* frameshift mutation identified in this study, p.(N388Rfs*20), is located in exon 3 (indicated by †). (**b**) Schematic of *GCNT2* genomic structure with black bars representing coding exons (not to scale). Three alternatively transcribed exon 1 (1A, 1B and 1C) indicate exons used in different *GCNT2* isoforms.^10,16^ (**c**) Three *GCNT2* isoforms designated *GCNT2-A*, *-B* and *-C* result from alternative transcribed exon 1, but identical exon 2 and 3.^10,16^ The size of each protein isoform is also shown.

**Table 1:** Summary of *GCNT2* mutations identified and resulting phenotypes

*No*	*Homo/Comp. Het*	*Nucleotide change*	*Protein change*	*Location (Exon)*	*Ethnicity*	*Clinical features*		*Ref.*	*Polyphen2 (human variation score 0 to 1)*	*SIFT (tolerance index 0 to 1)*	*Blosum 62 score (−4 to 11)*	*ExAC total individuals (heterozygous or homozygous^†^)*
						*Adult i blood group*	*Cataract*					
1	Homo	c.1043G>A/c.1043G>A	p.(G348E)/p.(G348E)	3	Taiwanese (1 family)	Yes	Yes	15	POS (0.548)	DMG (0)	−2	9/60 694
2	Comp. Het	c.1043G>A/c.1148G>A	p.(G348E)/p.(R383H)	3 3	Taiwanese (1 family)	Yes	Yes	15	POS (0.548) BNG (0.037)	DMG (0) TOL (0.08)	−2 0	9/60 694; 5/60 682
3	Homo	Segmental deletion (deletion encompassing exons 1B, 1C, 2 and 3)	No protein	NA	Taiwanese (1 family)	Yes	Yes	15	NA	NA	NA	NA
4	Homo	c.505G>A/c.505G>A	p.(A169T)0/p.(A169T)	1C 1C	White (5 unrelated patients)	Yes	No	10	PRD (0.990)	DMG (0)	0	572/60 656 (1/60 656^†^)
5	Comp. Het	c.505G>A/c.683G>A	p.(A169T)/p.(R226Q)	1C 1C	White (1 patient)	Yes	No	10	PRD (0.990) PRD (0.998)	DMG (0) DMG (0)	0 1	572/60 656 (1/60 656^†^); 2/60 675
6	Comp. Het	c.1000G>A/c.1043G>A	p.(G334R)/p.(G348E)	2 3	Japanese (1 family)	Yes	Yes	16	POS (0.745) POS (0.548)	TOL (0.51) DMG (0)	−2 −2	1/57 697; 9/60 694
7	Homo	c.977G>A/c.977G>A	p.(W326*)/p.(W326*)	2	Arabic (4 families)	Yes	Yes	17	NA	NA	NA	0/58 867
8	Homo	c.929G>A/c.929G>A/	p.(G310D)/ p.(G310D)	2	Persian Jews (1 family)	Yes	Yes	18	POS (0.798)	DMG (0.05)	−1	2/54 924
9	Homo	Segmental deletion (~93 kb deletion encompassing exons 1B, 1C, 2 and 3)	No protein	NA	Pakistani (2 families)	Yes	Yes	19	NA	NA	NA	NA
**10**	**Homo**	**c.1163_1166delATCA/c.1163_1166delATCA**	p.(N388Rfs*20)/p.(N388Rfs*20)	**3**	**White (1 family)**	**Yes**	**Yes**	**This study**	**NA**	**NA**	**NA**	**0/60 675**

Abbreviations: BNG, benign; Comp. het, compound heterozygous; DMG, damaging; ExAC, Exome Aggregation Consortium; Homo, homozygous; NA, not available; PRD, probably damaging; POS, possibly damaging; TOL, tolerated.

All variant annotations are numbered in accordance with *GCNT2-B* (Ensembl transcript ID: ENST00000316170), except p.(A169T) and p.(R226Q) variants, which are numbered according to *GCNT2-C* (Ensembl transcript ID: ENST00000265012) due to their locations in *GCNT2-C-*specific exon 1C (Figure 2). For each mutation, ethnicity and number of the reported families are shown. The clinical features describe the presence or absence of adult i blood group and cataracts in the affecteds. *In silico* analysis of *GCNT2* mutations identified is presented. Polyphen2 appraises mutations quantitatively as benign, possibly damaging or probably damaging based on the model’s false-positive ratio. SIFT results are reported to be tolerated if tolerance index is ⩾0.05 or damaging if tolerance index is <0.05. Blosum 62 substitution matrix score; positive numbers indicate a substitution more likely to be tolerated evolutionarily and negative numbers suggest the opposite. ExAC denotes variants in the Exome Aggregation Consortium database (accessed 10 July 2016). The frequency of each variant contributing to the compound heterozygous mutation is separated by a semicolon ‘;’. None of the patient variants were identified in a homozygous state in the control population consisting of 60 656 individuals, except variant p.(A169T), indicated by †, in which one European individual from the control population was reported to be homozygous for the variant. The mutation reported in this study is highlighted in bold.
